# Bidirectional Mendelian randomization links gut microbiota to primary biliary cholangitis

**DOI:** 10.1038/s41598-024-79227-z

**Published:** 2024-11-16

**Authors:** Zhijia Zhou, Wenxuan Li, Yuelan Wu, Tao Wang, Jinghao Zhang, Liping You, Haoran Li, Chao Zheng, Yueqiu Gao, Xuehua Sun

**Affiliations:** 1 Department of Hepatology, ShuGuang Hospital Affiliated to Shanghai University of Traditional Chinese Medicine, Shanghai, 201203 China; 2 Central Laboratory, ShuGuang Hospital Affiliated to Shanghai University of Traditional Chinese Medicine, Shanghai, China; 3 Shanghai University of Traditional Chinese Medicine, Shanghai, China

**Keywords:** Primary biliary cholangitis, Gut microbiota, Mendelian randomization, Bidirectional, Causal relationship, Clinical microbiology, Biliary tract disease

## Abstract

Primary biliary cholangitis (PBC) and gut microbiota (GM) are epidemiologically correlated but the causal inter-relationships remain poorly understood. We aim to explore the causal relationships between GM and PBC. Using the MiBioGen consortium, GWAS data for GM at the species level and the largest publicly available PBC GWAS data to date, we performed a bidirectional two-sample Mendelian randomization by the inverse variance weighted, MR-Egger, weighted median, weighted model and MR-PRESSO to elucidate the potential causal role of GM in PBC. To measure the heterogeneity of instrumental variables (IV), Cochran’s Q statistic and MR-Egger intercept test were used. Genetically instrumented order *Coriobacteriales* (odds ratio [OR] = 2.18, 95% confidence interval [CI] 1.30–3.66, *P* = 0.004) significantly increased the risk for PBC, while genetically driven class *Deltaproteobacteria* (OR = 0.52, 95% CI 0.36–0.74, *P* = 0.002) causally decrease the NAFLD risk. Reverse MR analysis showed no significant association between PBC and the two specific GM. However, it indicated that PBC progression significantly increases the abundance of the class *Bacteroidia*, order *Bacteroidales*, and phylum *Bacteroidetes* (OR = 1.02, 95% CI 1.002–1.03, *P* = 0.026), while decreasing the abundance of the genus *Lachnospiraceae UCG010* (OR = 0.98, 95% CI 0.96–0.995, *P* = 0.026). Our study demonstrated that genetically driven order *Coriobacteriales* and class *Deltaproteobacteria* were causally related to PBC risk. This causality provided a new perspective on ameliorating PBC by modulating GM. Our study demonstrated that genetically driven order *Coriobacteriales* and class *Deltaproteobacteria* were causally related to PBC risk. PBC was causally related to the abundance of four GM taxa(class *Bacteroidia*, order *Bacteroidales*, phylum *Bacteroidetes* and genus *Lachnospiraceae* UCG010). This causality provided a new perspective on ameliorating PBC by modulating GM.

**Supplementary Information:**

The online version contains supplementary material available at 10.1038/s41598-024-79227-z.

## Introduction

Primary biliary cholangitis (PBC) is characterized by chronic, non-suppurative, lymphocytic destructive cholangitis, the presence of specific anti-mitochondrial antibodies (AMAs), and a female predominance^1^. It is distributed globally, with its incidence and prevalence on the rise worldwide^2^. Despite its typically slow progression, PBC has a detrimental impact on the overall health of patients, leading to liver fibrosis and eventual cirrhosis^3^. To date, the pathophysiology of PBC remains incompletely understood, with genetic susceptibility, immune dysregulation, and environmental risk factors believed to play a collective role in its development^4,5^.

The gut microbiota(GM) consists of a diverse array of bacteria, archaea, fungi, and viruses, participating in various crucial metabolic and immune processes, including host immune regulation, food digestion, gut endocrine function, and intestinal permeability^6,7^. The microbiome exerts its influence upon the host organism by modulating the host immune system, facilitating host physiological processes through the generation or metabolism of endogenous compounds, and catalyzing the transformation of exogenous substances, encompassing nutrients and pharmaceutical agents^8^. Notably, gut dysbiosis is recognized as a significant contributor to autoimmune liver diseases such as Primary Sclerosing Cholangitis (PSC) and PBC^9,10^. Dysbiosis of the GM promotes hepatobiliary injury in PBC patients through multiple mechanisms, including affecting intestinal mucosal immune homeostasis^11^, increasing intestinal permeability and thereby promoting bacterial translocation, inducing aberrant immune activation through the LPS/TLR4 signaling pathway^12^ and molecular mimicry mechanisms^13^, and inhibiting microbial bile acid(BA) metabolism.

In the current therapeutic landscape for PBC, the primary pharmacological intervention entails the utilization of ursodeoxycholic acid (UDCA), a bile acid metabolism modulator. However, approximately 40% of patients do not respond well to this treatment. Research has indicated significant differences in GM between responders and non-responders to UDCA, suggesting that the intestinal microenvironment may play a crucial role in determining the response to UDCA^14^. Increasing research has highlighted the significant role of the GM in the pathogenesis of PBC, making interventions targeting the microbiota a promising avenue for new diagnostics and treatments^10,15^. However, the relationship between the microbial community and liver diseases is bidirectional and varies throughout the disease process. This makes it challenging to determine whether these microbial community changes are initiating or driving factors in autoimmune liver diseases, or if they are simply side effects or alterations in the clinical course due to the disease and/or medical interventions. The etiological relationship between alterations in GM and the onset of pathological conditions remains presently elusive. Consequently, there is a pressing demand for novel and pioneering methodologies to elucidate the causative involvement of the GM in the pathogenesis of human diseases.

Mendelian randomization (MR) is a method that utilizes genetic variants as instrumental variables to assess the causal effects of risk factors on outcomes^16^. It is less susceptible to bias from extensive confounding factors encountered in multivariable observational analyses and is less affected by measurement errors^17^. Moreover, owing to the immutable nature of genetic variation established at the point of conception, two-sample Mendelian randomization (TSMR) is less susceptible to the confounding effects of reverse causality bias. Consequently, over the past decade, MR has garnered escalating scrutiny as a robust and dependable approach for investigating causal associations between diverse risk factors and a spectrum of health outcomes^18^. When both types of evidence are available, the results of MR are very close to those of randomized controlled trials (RCTs).

To date, there are no validated longitudinal cohort studies or RCTs to elucidate the potential causal relationship between GM and PBC. In the present study, we aimed to elucidate the potential causal role of GM in PBC via a bidirectional two-sample Mendelian Randomization.

## Methods

### Assumptions and design

**Fig. 1:**
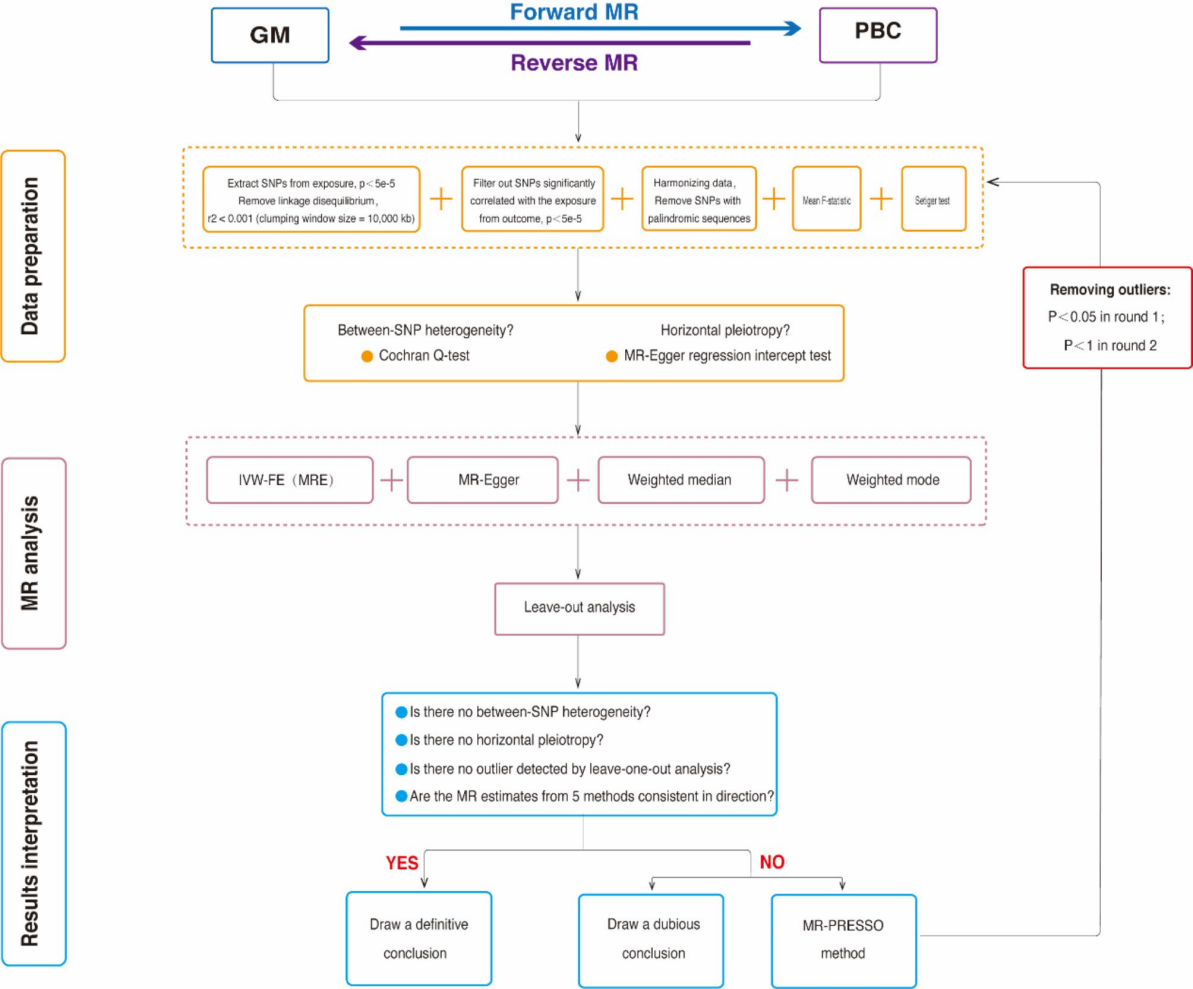
The conceptual framework and research roadmap of current research.

Figure 1 illustrates the flowchart of the TSMR study between GM and PBC. our MR study adhered to three key assumptions: (1) GM taxa under investigation had a close connection with the instrumental variables (IVs) employed in the analysis; (2) There was no evidence of interdependence between the selected IVs and confounding factors that might influence both GM taxa and the development of PBC; (3) Horizontal pleiotropy was carefully considered and ruled out, as any impact of the IVs on PBC was exclusively mediated through their influence on GM taxa. Our research results were disseminated in compliance with the MRSTROBE(Mendelian Randomization-Strengthening the Reporting of Observational Studies in Epidemiology) guidelines(table S8), as recommended in the field^19^.

### GWAS data

For GM and PBC, summary-level data from the genome-wide association study (GWAS) was acquired. The MiBioGen study is the largest genome-wide meta-analysis of ethnic diversity ever conducted on transgenics. Genotype and 16s fecal microbiome data were collected from 18,340 individuals from 24 different study populations^20^. Meanwhile, data on PBC were obtained from a meta-analysis of 8,021 cases and 16,489 controls of European descent, diagnosed using the International Classification of Diseases (ICD-10) criteria(GCST90061440 for PBC)^5^. The summary statistics for GM and PBC do not contain any personal information, and the GWAS have obtained ethical approval from relevant ethics review boards.

### Instrument variable selection

Instrumental variables (IVs) were selected based on rigorous criteria as follows: (1) Potential IVs were identified from single nucleotide polymorphisms (SNPs) associated with each species at a significance level of (*P* = 1 × 10^−5^)0.2) Linkage disequilibrium (LD) between SNPs was calculated using data from European samples within the 1000 Genomes Project as the reference genome. Only SNPs with the lowest *P*-value among those with r^2^ < 0.001 were retained, and this was achieved using a linkage window size of 10,000 kb. (3) SNPs associated with exposure for each outcome were extracted at a significance level of (*P* = 1 × 10^−5^). SNPs for which exposure-related SNPs were not available were excluded from the analysis. (4) Following the harmonization of exposure and outcome SNPs, the analysis further excluded palindromic SNPs, outliers identified through MR multifaceted residuals and MR-PRESSO global tests, as well as SNPs with a minor allele frequency of less than 0.01 and an F statistic (F = (beta/se) ^2^) < 10.

### Statistical analysis

In this study, a variety of methodologies were employed to ascertain the potential causal relationship between the GM and PBC. These methodologies encompassed inverse variance weighted (IVW), MR-Egger, weighted median, weighted modeling, and MR-PRESSO. To assess the robustness of key findings, additional examinations for heterogeneity and horizontal pleiotropy were conducted using meta-analytical techniques. These assessments involved the utilization of the modified Cochran Q statistic and the MR Egger intercept bias test. To mitigate potential horizontal pleiotropy introduced by individual SNPs, a systematic leave-one-out analysis was conducted, systematically excluding one SNP at a time from the analysis. Steiger’s test was implemented to investigate the presence of reverse causality. The analytical procedures were executed using R packages TwoSampleMR and MRPRESSO version 4.2.1. For data visualization, the R package ggplot2 was employed. At each level (phylum, order, order, family, and genus), we set a multiple-test significance threshold of *P* < 0.05/n (n is the effective number of independent bacterial taxa at the corresponding classification level)^21^.

## Result

### The causal effect of GM on PBC via forward MR

**Fig. 2:**
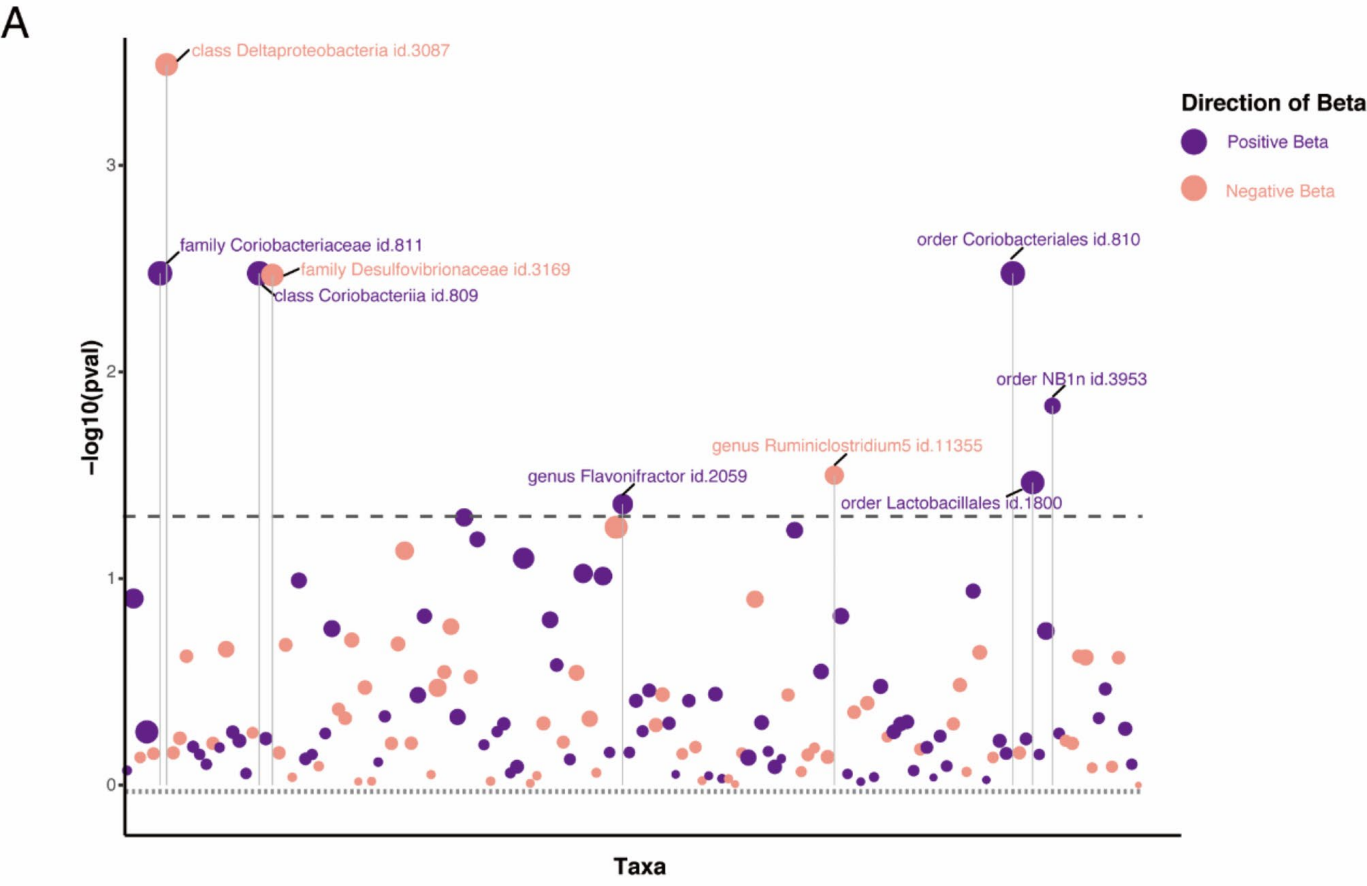
Lollipop plot was constructed to illustrate the outcomes of the IVW analysis concerning the impact of 196 gut microbiota (GM) taxa on primary biliary cholangitis (PBC). In this plot, positive beta values are depicted in purple, while negative beta values are represented in pink. Dashed lines positioned above the plot indicate p-values below the 0.05 threshold. Taxa that achieved statistical significance are explicitly labeled in the plot.

After excluding 15 unknown classifications, we analyzed 196 GM taxa, which is presented in the lollipop plot in Fig. 1. Detailed information on the 196 taxa is provided in Table S1. The genus *Ruminiclostridium* and order *NB1n* were excluded from the analysis due to disparate effect directions observed in MR-Egger compared to the other three methods. Furthermore, we removed the instrumental variable rs2952251 from the order *Lactobacillales* because of its association with the confounder using PhenoScanner V2(www.phenoscanner.medschl.cam.ac.uk)^22^ (Table S3). Following the procedural steps delineated in Fig. 1, a total of six GM taxa that met the defined criteria were ultimately identified (Fig. 2).

**Fig. 3:**
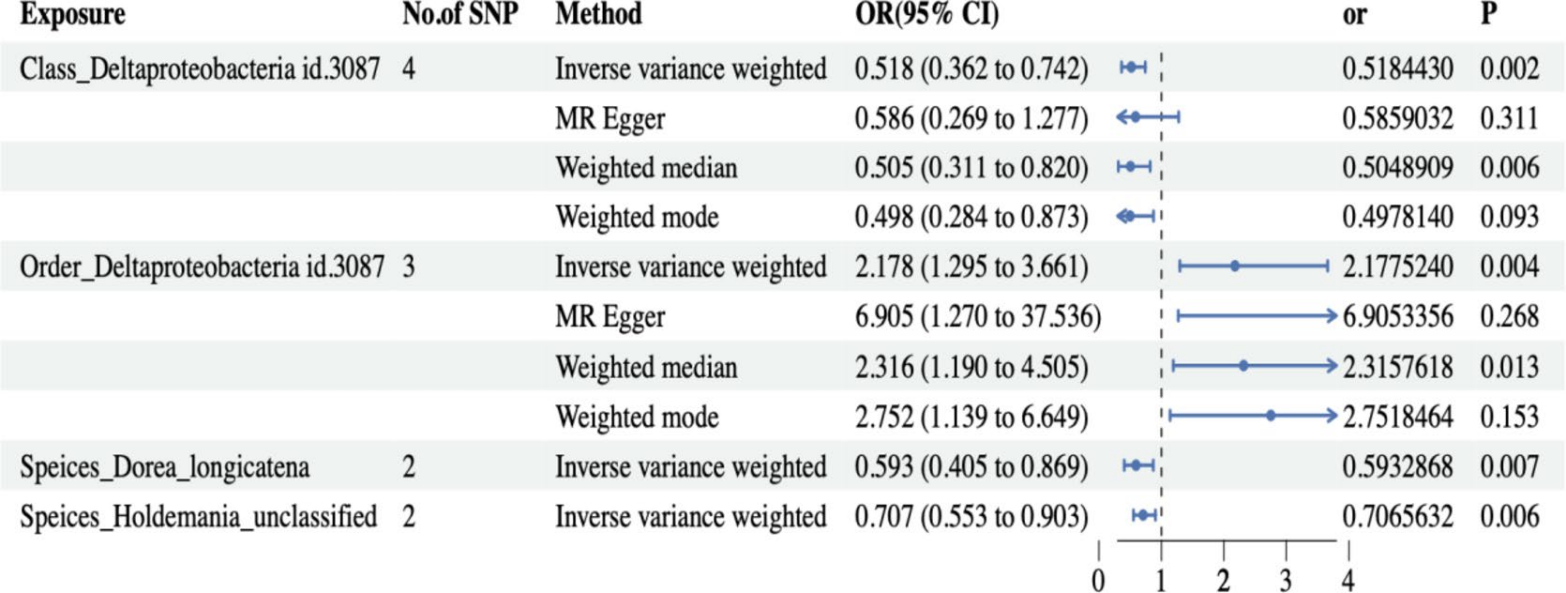
Forest plot presenting the results of four analyses on the genetic associations between gut microbiota (GM) and primary biliary cholangitis (PBC). The *P-*values of the IVW method have been adjusted using false discovery rate (FDR) correction. Traits with less than 3 SNPs can only be analyzed by the IVW method.

Results from the IVW analysis revealed that high abundance of class *Coriobacteriia*, family *Coriobacteriaceae*, order *Coriobacteriales* (odds ratio [OR] = 2.18, 95% confidence interval [CI] 1.30–3.66, *P* = 0.004), and genus *Flavonifractor* (OR = 1.61, 95% CI 1.01–2.56, *P* = 0.044) was associated with higher PBC risks. Conversely, a high abundance of class *Deltaproteobacteria* (OR = 0.52, 95% CI 0.36–0.74, *P* = 0.002) and family *Desulfovibrionaceae* (OR = 0.53, 95% CI 0.34–0.81, *P* = 0.004) was associated with lower PBC risks. Weighted median MR estimates indicate that class *Coriobacteriia* (OR = 2.32, 95% CI 1.18–4.56, *P* = 0.015), family *Coriobacteriaceae* (OR = 2.32, 95% CI 1.19–4.51, *P* = 0.014), and order *Coriobacteriales* (OR = 2.32, 95% CI 1.19–4.50, *P* = 0.013) are risk factors for PBC. Conversely, class *Deltaproteobacteria* (OR = 0.50, 95% CI 0.31–0.82, *P* = 0.006) and family *Desulfovibrionaceae* (OR = 0.51, 95% CI 0.31–0.85, *P* = 0.009) serve as protective factors for PBC (Fig. 3 and Table S3). above *p*-values of the IVW method have been adjusted using false discovery rate (FDR) correction.

To further mitigate the impact of potential false-positive results on our conclusions, we applied Bonferroni multiple testing correction. Specifically, the MR analysis identified a total of 149 distinct bacterial taxa. This included 88 taxa at the genus level (with a significance threshold of *P* < 5.68 × 10^− 4^, calculated as 0.05/88), 27 at the family level (*P* < 1.85 × 10^− 3^, based on 0.05/27), 14 at the order level (*P* < 3.57 × 10^− 3^, with a threshold of 0.05/14), 12 at the class level (*P* < 4.17 × 10^− 3^, corresponding to 0.05/12), and eight at the phylum level (*P* < 6.25 × 10^− 3^, derived from 0.05/8).After applying Bonferroni correction, only the order *Coriobacteriales* (OR = 2.18, 95% CI 1.30–3.66, *P* = 0.004) and the class *Deltaproteobacteria* (OR = 0.52, 95% CI 0.36–0.74, *P* = 0.002) showed a causal relationship with PBC. For details on significant classification SNPs, refer to Table S2.

Due to the lack of species-level data in the MiBioGen study database, we utilized data from a GWAS cohort study conducted in the Netherlands^23^. Following the criteria for IV selection described previously, we performed clumping and used PhenoScanner V2. The F-statistics of all IVs were greater than 10, indicating no evidence of weak instrument bias. Three pleiotropic SNPs were removed using the MR-PRESSO outlier test. After harmonizing the exposure and outcome data, a total of 959 IVs remained, covering 70 microbiome species (Table S9). In the forward MR analysis, two species, *Dorea longicatena* (OR = -0.52, 95% CI: -0.90 to -0.14, *P* = 0.007) and *Holdemania* (unclassified) (OR = -0.34, 95% CI: -0.59 to -0.10, *P* = 0.005), were found to PBC using the IVW method, as shown in Fig.3 (Refer to table S10 for results for other species). However, after Bonferroni multiple testing correction, the significance threshold was set at *P* < 0.05/70 = 7.14 × 10^− 4^, indicating that the observed associations were not statistically significant.

Lastly, we conducted assessments for both heterogeneity and horizontal pleiotropy. The findings from Cochran’s Q statistics revealed an absence of heterogeneity among individual SNPs. Furthermore, the results from MR-Egger regression and MR-PRESSO global tests provided indications that horizontal pleiotropy was unlikely to introduce bias into the observed effect of GM on PBC, as detailed in Table S4. Leave-one-out analysis demonstrated that the inferred causal relationship between GM and PBC could not be attributed to the influence of a single SNP. These estimations are visually presented in Figure S1.

### The causal effect of PBC on GM via reverse MR

**Fig. 4:**
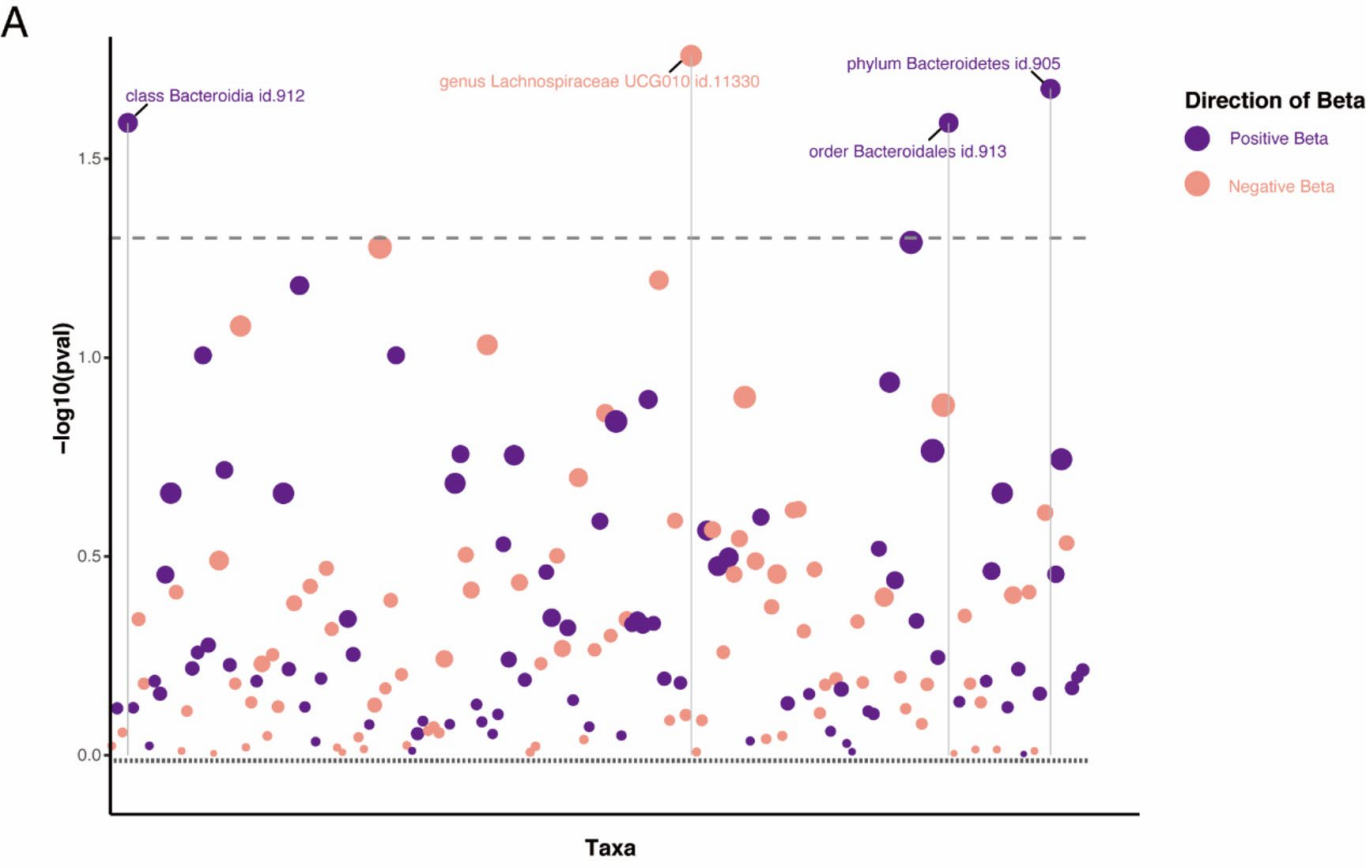
A lollipop plot was constructed to illustrate the outcomes of the IVW analysis concerning the impact of primary biliary cholangitis (PBC) on 196 gut microbiota (GM) taxa. Positive beta values are depicted in purple, while negative beta values are represented in pink. Dashed lines positioned above the plot indicate p-values below the 0.05 threshold. Taxa that achieved statistical significance are explicitly labeled in the plot.

**Fig. 5:**
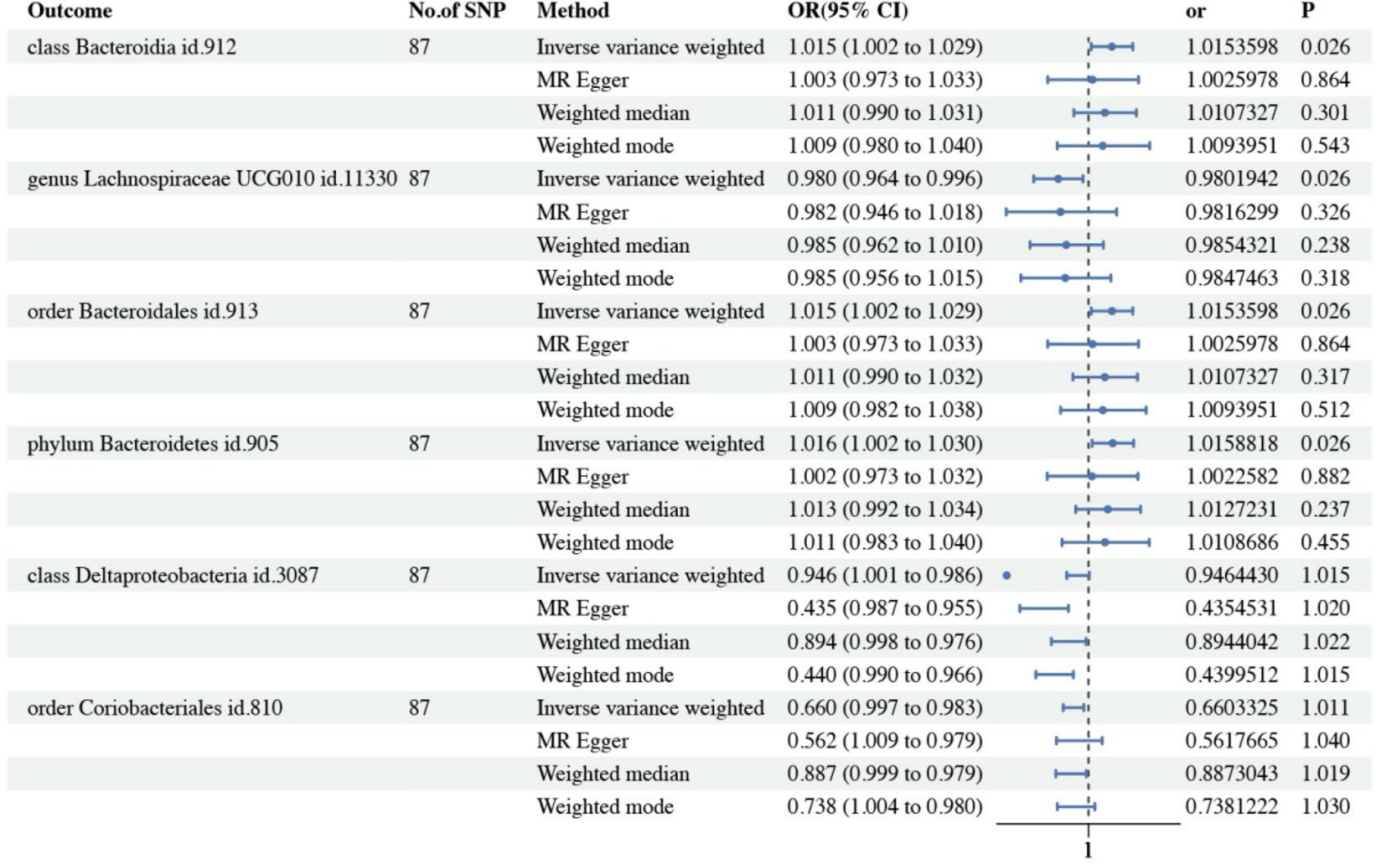
Forest plot presenting the results of four analyses on the genetic associations between primary biliary cholangitis (PBC) and gut microbiota (GM). The *P*-values of the IVW method have been adjusted using false discovery rate (FDR) correction. Traits with less than 3 SNPs can only be analyzed by the IVW method.

To further investigate whether PBC leads to dysbiosis or if there exists a reverse causal relationship, reverse MR analysis was performed according to the selection criteria for IVs, we identified 87 SNPs associated with PBC. The IVW analysis for PBC on 196 GM taxa is depicted in the lollipop plot in Fig. 4. The results of IVW analysis indicate that progress of PBC significantly higher the abundance of class *Bacteroidia*, order *Bacteroidales*, phylum *Bacteroidetes* (OR = 1.02, 95% CI 1.002–1.03, *P* = 0.026), and lower the abundance of genus *Lachnospiraceae* UCG010 (OR = 0.98, 95% CI 0.96–0.995, *P* = 0.026), Importantly, PBC did not show a significant causal association with the order *Coriobacteriales* or the class *Deltaproteobacteria* (Fig. 5 and Table S6). The above *p*-values of the IVW method have been adjusted using false discovery rate (FDR) correction. Significant taxonomic SNPs are presented in Table S5. Likely, we conducted assessments for both heterogeneity and horizontal pleiotropy. The findings from Cochran’s Q statistics revealed an absence of heterogeneity among individual SNPs. Furthermore, the results from MR-Egger regression and MR-PRESSO global tests provided indications that horizontal pleiotropy was unlikely to introduce bias into the observed effect of PBC on GM taxa, as detailed in Table S4. Leave-one-out analysis demonstrated that the inferred causal relationship between PBC and GM taxa could not be attributed to the influence of a single SNP. These estimations are visually presented in Figure S2.Heterogeneity tests revealed no significant heterogeneity among individual SNPs. Based on the results of MR-Egger regression and MR-PRESSO global tests (Table S7), it appears less likely that horizontal pleiotropy distorts the impact of PBC on GM.

## Discussion

In the present study, we explored the causal association between gut flora at different levels and PBC using Mendelian randomization. Due to the lack of species-level data in the MiBioGen consortium, we integrated data from the study of Lopera-Maya et al.^23^. Thus, for the first time, we systematically explored possible associations GM and PBC from six categories: phylum, class, order, family, genus and species. The preliminary TSMR analysis has ascertained 12 GM taxa associated with PBC (Figs. 3, 5). Specifically, in the forward MR analysis, 8 GM taxa are causally associated with PBC, including class *Coriobacteriia*, order *Coriobacteriales*, family *Coriobacteriaceae*, genus *Flavonifractor*, class *Deltaproteobacteria*, family *Desulfovibrionaceae*, species *Dorea longicatena* and species *Holdemania* (unclassified). Conversely, in the reverse MR analysis, PBC does not endorse any causal relationships with the aforementioned microbial classifications. However, PBC influences the abundance of phylum *Bacteroidetes*, class *Bacteroidia*, order *Bacteroidales*, and genus *Lachnospiraceae* UCG010.

However, after rigorous screening for significance thresholds, our TSMR study demonstrated a causal relationship between the class *Deltaproteobacteria*, order *Coriobacteriales* and the development of PBC. We revealed that class *Deltaproteobacteria* may serve as protective factors against PBC. Class *Deltaproteobacteria* is sulfate-reducing bacteria (SRB), comprising the most known sulfur bacteria such as *Desulfurivibrio*, *Desulfomicrobium*, *Desulfococcus*, and others. *Deltaproteobacteria* play a crucial role in human food digestion, can accumulate various extracellular digestive enzymes, thereby enhancing the efficiency of food digestion within the gut^24^.Family *Desulfovibrionaceae*, a subgroup of class *Deltaproteobacteria*, constitutes an anaerobic, Gram-negative bacterial family that acts as an opportunistic pathogen and plays a significant role in various diseases, including autoimmune diseases^25,26^, infectious diseases^27,28^, and metabolic diseases^29^.In a systematic review, it was noted that PBC patients exhibit a lower abundance of the genus *Desulfovibrio* compared to healthy individuals^30^. Nevertheless, contradictory observations have also been reported, which could be influenced by the sample size limitations and other confounding factors inherent in observational studies. It is worth noting that the existing research on GM metagenomics predominantly stems from Asian patient populations, thus exhibiting certain limitations in terms of generalizability.However, in a recent large-scale cohort study on PBC, it was observed that *Desulfovibrionaceae* within the UDCA responder microbiota exhibited higher abundance compared to non-responders^14^. This suggests a close association between *Desulfovibrionaceae* and bile acid metabolism. *Desulfovibrionaceae* is a taxa that possesses bile salt hydrolases (BSHs), enzymes that catalyze the hydrolysis of amide bonds, leading to the dissociation of glycine-conjugated and taurine-conjugated bile acids, releasing free BA (such as cholic acid and deoxycholic acid) along with amino acids (such as glycine and taurine)^31^.The higher relative abundance of glycine-bound BAs and UDCA-bound taurine in the feces of UDCA responders suggests enhanced hepatic reabsorption and excretion capabilities, reflecting improvements in bile stasis and liver function^14^. Consequently, host metabolism can be influenced by microbial modifications of BAs, leading to alterations in immune signaling through bile acid receptors and changes in immune responses triggered by shifts in the microbial composition^15,32^. *Desulfovibrionaceae* generates hydrogen sulfide (H_2_S) as its ultimate byproduct^33^. However, some studies have shown that H_2_S inhibits TNF-α and IFN-γ induces functional damage to the intestinal epithelial barrier^34^. Not only that, H2S can also ameliorate dextran sulfate sodium (DSS)-induced intestinal barrier damage^35^. Given that some patients with early-stage PBC may experience barrier damage and increased permeability^36^, the protective effect of *Desulfovibrionaceae* on PBC may stem from the protective effect of its metabolites on the intestinal barrier. However, further studies are needed to verify this conjecture.

According to the TSMR analysis, we have identified that order *Coriobacteriales* was causative risk factors for PBC. It has been reported that in patients with acute myeloid leukemia (AML), there is a significant enrichment of *Coriobacteriia*, which is significantly positively correlated with hydroxyprolyl-hydroxyproline, prolyl-tyrosine, and tyrosyl-proline, amino acid derivatives^37^. Disruptions in amino acids and their derivative metabolism can affect various biological processes, including cell survival, immune responses, epigenetics, and redox regulation, and are associated with various diseases, including PBC. Therefore, we speculate that *Coriobacteriia* may promote the progression of PBC by influencing amino acid metabolism. However, there are no studies on the relationship between these bacterial taxa and PBC, and this study is the first to report a specific bacterial group associated with PBC. Therefore, our study may provide a new perspective for elucidating the link between *Coriobacteriia* and PBC.

In addition, the genus *Flavonifractor* is a bacterium capable of degrading flavonoids. It has been found to be increased in a variety of diseases^38^ and is strongly associated with increased oxidative stress and systemic low-grade inflammation^39^.The breakdown of quercetin by *Flavonifractor* may contribute to increased oxidative stress and inflammation^40^ and it has also been associated with the progression of colorectal cancer^41^. A recent MR study has also shown that the genus *Flavonifractor* is a risk factor for Irritable Bowel Syndrome^42^. Furthermore, a metagenomic sequencing study showed that *Flavonifractor* contains the bile acid 7-dehydroxylation gene, suggesting that excess may disrupt the homeostasis of bile acid metabolism^43^. Secondary bile acids can act as signaling molecules, interacting with intestinal cell receptors to regulate overall metabolism, which may subsequently impact the progression of PBC. Any disturbance in the GM and BAs can profoundly affect the body’s pathological and physiological processes^44^. The significant alterations in the GM composition and impaired microbial bile acid metabolism in PBC patients may be a contributing factor to the consequences of conditions such as bile stasis.

In contrast to the causal relationships established through forward MR analysis, the reverse Mendelian randomization findings do not lend support to the notion of bidirectional causality between the mentioned GM taxa and PBC. However, it is noteworthy that our investigation has revealed causal relationships of PBC on several GM taxa, including phylum *Bacteroidetes*, class *Bacteroidia*, order *Bacteroidales*, and the family *Lachnospiraceae* UCG010. It is well known that PBC affects the intestinal environment by inducing intestinal motility disorders, immune disorders, bile secretion defects and portal hypertension, which in turn affects the alteration of GM^10,45^.

As intestinal commensals, *Bacteroidetes* protect the gut from pathogens and provide nutrition to other microorganisms in the gut. It has been observed that PBC patients have fewer *Bacteroidetes* compared to healthy individuals. However, some studies have not found significant differences between the two groups^46^. *Lachnospiraceae* are important butyrate producers in the gut microbiota. Butyrate and other Short-chain fatty acids inhibit intestinal inflammation and maintain the intestinal barrier through different mechanisms and regulate intestinal motility. *Lachnospiraceae* has been found to be lower^47^, including specific genera like *Lachnobacterium* spp., which are also decreased^48^. Metagenomic sequencing of fecal samples from PBC patients has shown a depletion of *Lachnobacteriums*, which is consistent with our conclusions.

Previous studies have reported a causal relationship between PBC and GM^49,50^. After multiple Bonferroni corrections, our findings were consistent with those of Luo et al.^49^. for the order *Coriobacteriales*, class *Coriobacteriia*, and class *Deltaproteobacteria*. However, after further FDR adjustments, we excluded *class Coriobacteriia*. Zhang et al.^50^ using the Cordell et al.^5^. PBC dataset (comprising 2,764 cases and 10,475 controls of European origin), identified five causal relationships involving the GM taxa: Order *Selenomonadales*, Order *Bifidobacteriales*, Genus *Lachnospiraceae_UCG_004*, Family *Peptostreptococcaceae*, and Family *Ruminococcaceae*. This may be since differences in PBC data sources influenced the IV selection and results. In addition, the PBC GWAS dataset used by Zhang et al. was relatively small and was not Bonferroni corrected, which could have led to potential false positives.

Overall, this study possesses several notable strengths. this is the first Mendelian randomization study to systematically explore the possible link between GM and PBC in six categories: phylum, order, order, family, genus and species. Moreover, the application of MR effectively mitigated the impact of reverse causality and potential confounding factors, facilitating the inference GM of causal effects between GM and PBC traits. SNPs employed for both GM and PBC were sourced from the largest available GWAS meta-analyses, ensuring robust IVs strength and result robustness. The reliability of causal effect estimates was further ensured through the utilization of various statistical models, such as inverse variance weighted, MR-Egger, weighted median, weighted model and MR-PRESSO, and, along with sensitivity analyses, including Cochran’s Q statistics and MR Egger intercept test. Our TSMR analysis pinpointed two bacterial taxa associated with PBC, which can serve as candidate microbial interventions for future clinical trials related to PBC. Additionally, our findings offer an innovative perspective on PBC research, suggesting the potential for targeted modulation of specific bacterial taxa, such as promoting beneficial bacteria and inhibiting harmful bacterial growth, for the prevention and treatment of PBC.

However, our study is not without limitations. First, the SNPs used in the forward MR analyses did not reach the conventional GWAS significance threshold (*P* < 5e-8). Nevertheless, the risk of false-positive results was significantly reduced by applying Bonferroni multiple testing correction and the FDR test. Second, the participants in the genomic meta-analyses of GM and PBC were predominantly of European ancestry. As a result, the same genetic variants may exhibit different pleiotropic effects in diverse ethnic groups, which suggests that the causal inferences drawn from our study may not be generalizable to non-European populations. Third, in the reverse MR analysis, despite using the largest PBC dataset to date, the sample size of the GWAS meta-analysis for PBC-related traits remains limited, potentially introducing bias due to weak IVs. Fourth, although we applied numerous exclusion criteria in selecting IVs and utilized tools such as PhenoScanner to eliminate IVs with potential pleiotropy, various intrinsic and extrinsic factors can influence both GM and PBC. Therefore, we cannot completely rule out the possibility of bias arising from the correlation of SNPs with potential confounding factors. Lastly, the absence of demographic data, such as sex, in the original studies precluded us from conducting subgroup analyses based on these characteristics. Our findings require further validation through clinical and mechanistic studies. Nonetheless, these results do not preclude the potential for interactions among these effects. A deeper investigation into these mechanisms will provide valuable insights into the pathogenesis of PBC and help identify alternative therapeutic targets to reduce the current dependence on UDCA treatment.

## Conclusion

In summary, this study represents the comprehensive exploration of causal relationships between GM and PBC using bidirectional MR analysis. Our study demonstrated that genetically driven order *Coriobacteriales* and class *Deltaproteobacteria* were causally related to PBC risk, and that PBC was causally related to the abundance of four GM taxa. These results substantiate the existence of causal relationships between PBC and GM, providing a foundation for techniques aimed at altering PBC through the modulation of GM.

## Electronic supplementary material

Below is the link to the electronic supplementary material.

Supplementary Material 1

## Data Availability

Publicly available datasets were analyzed in this study. This data can be found here: Gut microbiota GWAS data is from MiBioGen (https://mibiogen.gcc.rug.nl/). PBC GWAS data is from IEU OPEN gwas project release (https://gwas.mrcieu.ac.uk/).

## Abbreviations

PBC: Primary biliary cholangitis
GM: Gut microbiota
IV: Instrumental variables
OR: Odds ratio
CI: Confidence interval
AMAs: Anti-mitochondrial antibodies
PSC: Primary Sclerosing Cholangitis
UDCA: Ursodeoxycholic acid
MR: Mendelian randomization
RCTs: Randomized controlled trials
SNPs: Single nucleotide polymorphisms
LD: Linkage disequilibrium
IVW: Inverse variance weighted
FDR: False discovery rate
GAWS: Genome-wide association study
TSMR: Two-sample Mendelian randomization
SRB: Sulfate-reducing bacteria
BSHs: Bile salt hydrolases
BA: Bile acid
H_2_S: Hydrogen sulfide
DSS: Dextran sulfate sodium
AML: Acute myeloid leukemia
