# Supplementary figures and images for: Bidirectional Mendelian randomization links gut microbiota to primary biliary cholangitis
Source: Sci Rep. 2024 Nov 16;14:28301. doi: 10.1038/s41598-024-79227-z (PMC11569131; doi:10.1038/s41598-024-79227-z)

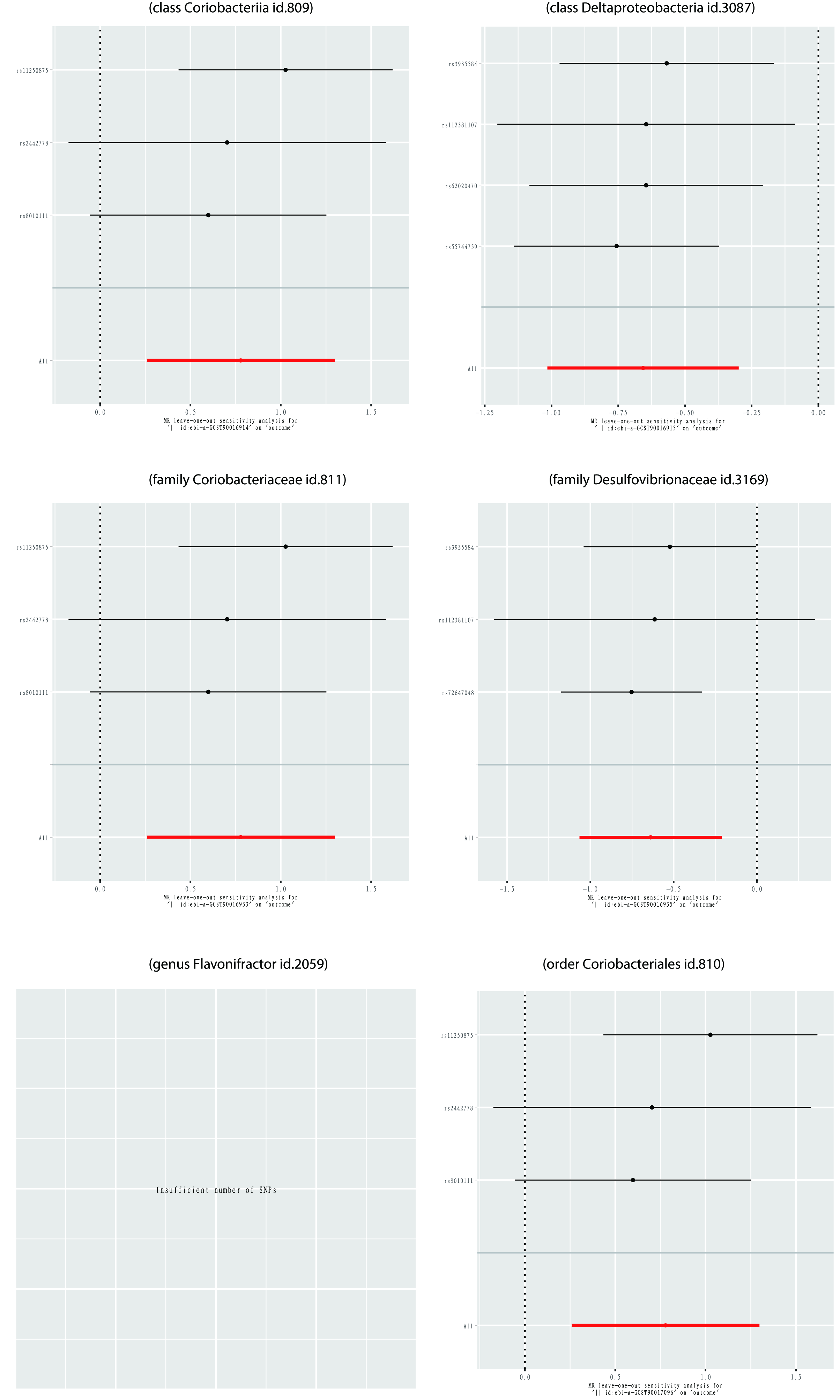

Supplement: Supplementary file 1 — Supplementary Material 1 [file 41598_2024_79227_MOESM1_ESM.zip › supplement_files/figureS1.tif]

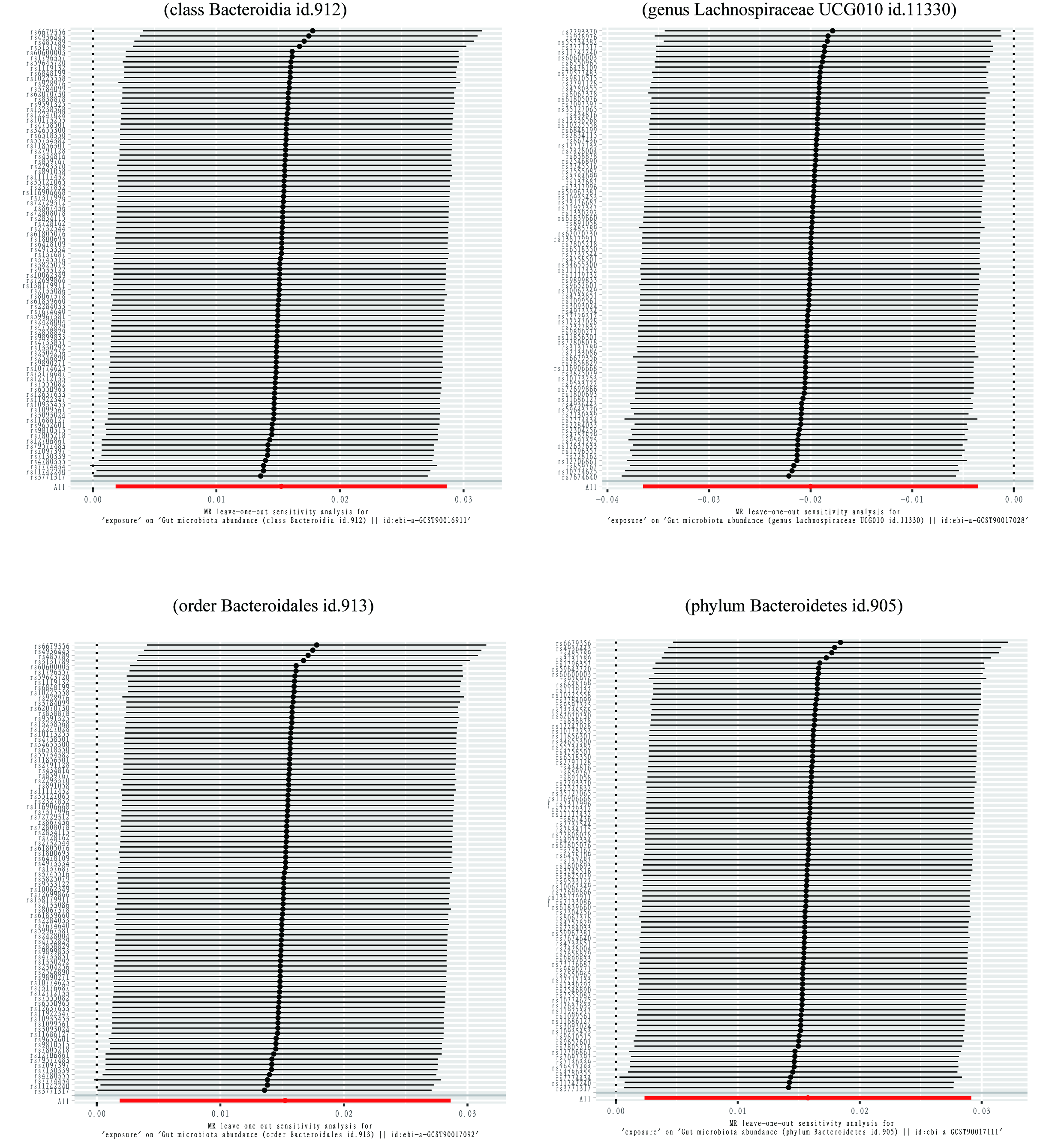

Supplement: Supplementary file 1 — Supplementary Material 1 [file 41598_2024_79227_MOESM1_ESM.zip › supplement_files/figureS2.tif]
